# Antioxidant activity of polysaccharide from *Garcinia mangostana* rind and their derivatives

**DOI:** 10.1186/s12906-024-04594-z

**Published:** 2024-07-25

**Authors:** Zhenjie Tang, Gangliang Huang

**Affiliations:** Key Laboratory of Carbohydrate Science and Engineering, Chongqing Normal University, Chongqing, 401331 China

**Keywords:** *Garcinia mangostana* rind polysaccharide, Chemical modification, Antioxidant activity

## Abstract

**Background:**

Polysaccharide from *Garcinia mangostana* rind has many biological activities and deserves further research.

**Methods:**

The antioxidant properties of UAEE-GMRP, UAEE-GMRP-1 A, CM-30, and Ac-30 were evaluated through two different antioxidant activity experimental systems.

**Results:**

The four polysaccharides had a better scavenging effect on hydroxyl radicals, while their inhibitory effect on lipid peroxidation was relatively weak. However, overall, the four polysaccharides showed a certain degree of potential application in the two antioxidant experiments mentioned above, especially the chemically modified polysaccharides from *Garcinia mangostana* rind, which effectively improved their antioxidant activity. This also indicates that chemical modification is a better method to improve polysaccharide activity. In addition, in these two antioxidant exploration experiments, carboxymethylated polysaccharide showed stronger activity compared to the other three polysaccharides.

**Conclusion:**

The carboxymethylation modification may have great potential for application.

## Introduction

Oxidation is a process of breaking down substances and releasing energy inside or outside the body of an organism. This type of action is widely present in nature, and in chemistry, we view it as a series of chemical reactions accompanied by electron gain and loss. Among them, in living organisms, the oxidation of substances is usually a slow and gentle process that does not make organisms feel the sensation of substance oxidation [1–3]. For example, in the human body, glucose and other energy producing substances are oxidized and broken down, producing a chemical called adenosine triphosphate (ATP), which is the energy that the body can utilize; In biological systems, the oxidation of substances may be a rapid and intense reaction, also known as a combustion reaction. In addition, oxidation is also reflected in the slow reaction between substances and oxygen, during which slow heating but no luminescence may occur, such as metal corrosion and biological respiration. In short, oxidation is closely related to our daily life, and appropriate oxidation can help clean hair, promote wound healing, and whiten the skin. Of course, there are beneficial oxidative effects, and naturally there are also some oxidative effects that can cause great damage to organisms. For example, excessive oxidative stress may lead to adverse effects on the body. During the respiration process of human cells, highly active deoxygenated radicals, hydroxyl radicals, and superoxide anion radicals are produced. If these negative oxidative effects and the generated oxygen free radicals are not cleared in a timely manner, when they accumulate in excess, it is highly likely to cause aging and certain diseases in the human body [4–10]. Therefore, people have been searching for substances that can safely and effectively eliminate oxygen free radicals, and the safety, non-toxicity, and natural antioxidant properties of polysaccharides have gradually been studied and studied by researchers. In view of this, antioxidant activity has become one of the widely studied biological activities of polysaccharides and an important indicator for evaluating the activity value of polysaccharides [11, 12].

In the experiment of measuring the antioxidant activity of polysaccharides, the excellent antioxidant activity of polysaccharides is often evaluated from the aspects of free radical scavenging ability, anti-lipid peroxidation ability, and total reduction ability. However, it should be noted that there may be varying degrees of differences in the expression of the above abilities by active polysaccharides. In this chapter’s experiment, the scavenging ability of *Garcinia mangostana* rind polysaccharide and their derivatives on hydroxyl radicals and their inhibitory ability on lipid peroxidation were investigated.

## Materials and methods

### Materials and reagents

The *Garcinia mangostana* rind was purchased from the market (Chongqing, China). UAEE-GMRP, UAEE-GMRP-1 A, CM-30, and Ac-30 were self-made in the laboratory. The related reagents were purchased through commercial channels, all of which were analytical purity.

### Determination of hydroxyl radical scavenging ability

The specific steps for determining the hydroxyl radical scavenging activity of polysaccharides are as follows [13]. Firstly, mix the polysaccharides with distilled water and prepare a polysaccharide solution with a total concentration of 2 mg/mL. Then dilute with water to obtain different concentrations of the test solution with a total volume of 1 mL, with concentrations of 0.125 mg/mL, 0.25 mg/mL, 0.5 mg/mL, 1 mg/mL, and 2 mg/mL, respectively. Afterwards, add 1 mL of newly prepared FeSO_4_ solution (6 mmol/L), 1 mL of H_2_O_2_ solution (5 mmol/L), and 1 mL of salicylic acid solution (20 mmol/L) to different concentrations of polysaccharide solutions. Finally, heat the above mixed solutions in a water bath at 37 ℃ for 30 min. After heating, perform UV scanning within the range of 400 ~ 600 nm and record the absorbance value at 510 nm. Distilled water replaced polysaccharide solution as the blank group, and V_c_ replaced polysaccharide solution as the positive control group. The measurement experiments were repeated three times. The formula for calculating the clearance rate of polysaccharides on hydroxyl radicals is:

1 $${{\rm{S}}_{ \cdot {\rm{OH}}}}\,\left( \% \right)\, = \,\left[ {\left( {{{\rm{A}}_{\rm{0}}}\, - \,{{\rm{A}}_{\rm{1}}}} \right)\,{\rm{/}}\,{{\rm{A}}_{\rm{0}}}} \right]\, \times \,100$$

In the formula, A_0_ and A_1_ represent the absorbance of the system without and with added samples, respectively.

### Determination of lipid peroxidation inhibition ability

The specific steps for measuring the anti-lipid peroxidation activity of polysaccharides are as follows [14]. Simply put, 2 mL of polysaccharide solutions with different mass concentrations (0.125 mg/mL, 0.25 mg/mL, 0.5 mg/mL, 1 mg/mL, and 2 mg/mL) are first taken and placed in a test tube. Then, a volume of 1.8 mL of soy lecithin solution and 0.4 mL of newly prepared FeSO_4_ solution are sequentially added to them (with concentrations of 1 mg/mL and 10 mmol/L, respectively). Next, heat the above mixed solutions in a water bath at 37 ℃ for 30 min. After heating, add 1 mL of trichloroacetic acid solution and thiobarbituric acid solution in sequence (with mass concentrations of 20% and 0.8%, respectively). Finally, heat in a 15 min boiling water bath and centrifuge after the reaction to remove a small amount of sediment while retaining the supernatant. Perform UV scanning on the above supernatant within the range of 400 ~ 600 nm and record the absorbance values at 535 nm. The experiment was repeated three times with distilled water as the blank group and Vc as the positive control group. The formula for calculating the inhibition rate of polysaccharides on lipid peroxidation is:

2 $${{\rm{I}}_{ \cdot {\rm{LP}}}}\,\left( \% \right)\, = \,\left[ {\left( {{{\rm{B}}_{\rm{0}}}\, - \,{{\rm{B}}_{\rm{1}}}} \right)\,{\rm{/}}\,{{\rm{B}}_{\rm{0}}}} \right]\, \times \,100$$

In the formula, B_0_ and B_1_ represent the absorbance of the system without and with added samples, respectively.

## Results and discussion

### Analysis of hydroxyl radical scavenging ability

**Fig. 1:**
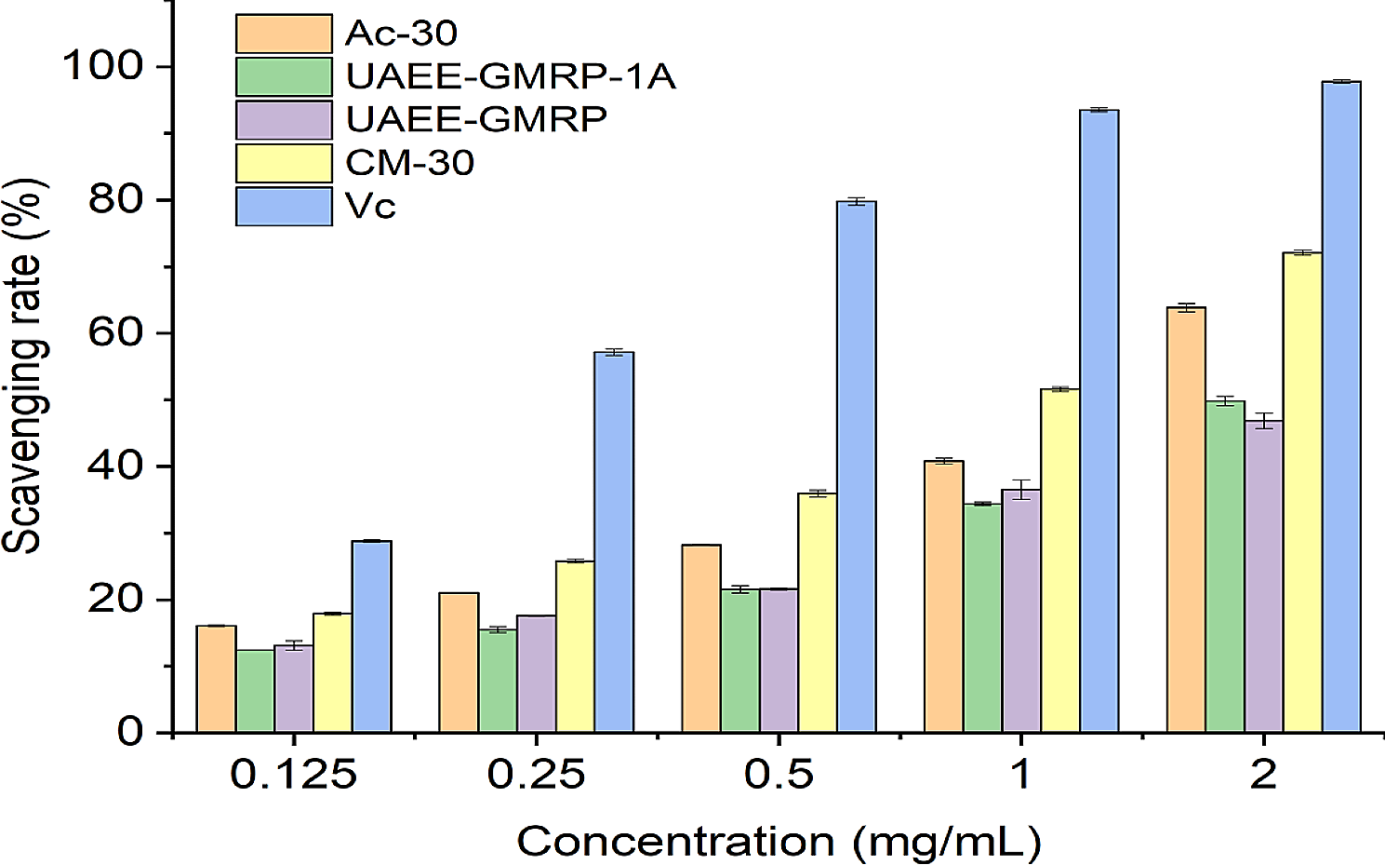
Hydroxyl radical scavenging ability

Hydroxyl radicals, as a highly oxidizing reactive oxygen species, have been widely proven to be closely related to human aging and the occurrence of certain diseases [15]. This part of the experiment used the Fenton reaction system to test the in vitro scavenging ability of four polysaccharides against hydroxyl radicals, and the relevant scavenging rate was used as an evaluation indicator. As shown in Fig. 1, it is evident that the clearance rates of the four polysaccharides are significantly positively correlated with their concentrations. After reaching the maximum concentration of 2 mg/mL, the clearance rates of UAEE-GMRP and UAEE-GMRP-1 A were basically half of those of the control group. At the same time, although the growth trend of the clearance rates of the two polysaccharides mentioned above has weakened to some extent compared to before after reaching a polysaccharide concentration of 1 mg/mL, there is still an upward trend. It is estimated that further increasing the concentration will still provide some room for improvement in their clearance ability. In addition, at various concentrations, the clearance rates of UAEE-GMRP and UAEE-GMRP-1 A are relatively similar, indicating that their ability to scavenge hydroxyl radicals mainly depends on the polysaccharide components. For Ac-30 and CM-30, it can be seen from the graph that their clearance rates are higher than the other two polysaccharides at the same concentration at all concentrations. After reaching the maximum concentration of 2 mg/mL, the clearance rate of CM-30 had reached three-quarters of that of the control group. Similarly, although the clearance rate of Ac-30 was lower than that of CM-30 at this concentration, it was still more than half of that of the control group. Furthermore, it is worth noting that according to the growth trend shown in the graph, it can be observed that after 2 mg/mL, CM-30 and Ac-30 have a better trend of improvement compared to the other two polysaccharides. Especially, the clearance rate of CM-30 has reached 72.4% at the maximum concentration. Further increase in concentration is likely to result in a situation where the clearance rate of V_c_ in the control group remains the same or even exceeds that of the control group. It can also be seen from here that chemical modification can indeed enhance the hydroxyl radical scavenging ability of polysaccharides, and the reason for this is likely due to the introduction of modification groups changing the spatial structure of polysaccharides, thereby enhancing their hydrogen supply ability and exposing more reaction sites [16, 17]. Of course, although chemical modification can enhance the biological activity of polysaccharides, the enhancement effects brought by different modification methods are not the same. For carboxymethylation modification, it is often similar to phosphorylation and sulfation, which can cause the aggregated polysaccharide molecules to stretch more to expose more hydrophilic hydroxyl groups, thereby significantly improving the water solubility of the modified polysaccharide [18]. The increase in aqueous solution can ensure the dissolution of more polysaccharide components to effectively exert their biological activity, which is also one of the reasons why the hydroxyl radical scavenging ability of CM-30 is generally higher than that of Ac-30 at various concentrations. In summary, all four polysaccharides can scavenge hydroxyl radicals to varying degrees, especially after undergoing carboxymethylation and acetylation modifications, their scavenging ability has been effectively improved, with carboxymethylation modification being the most significant.

### Analysis of lipid peroxidation inhibition ability

**Fig. 2:**
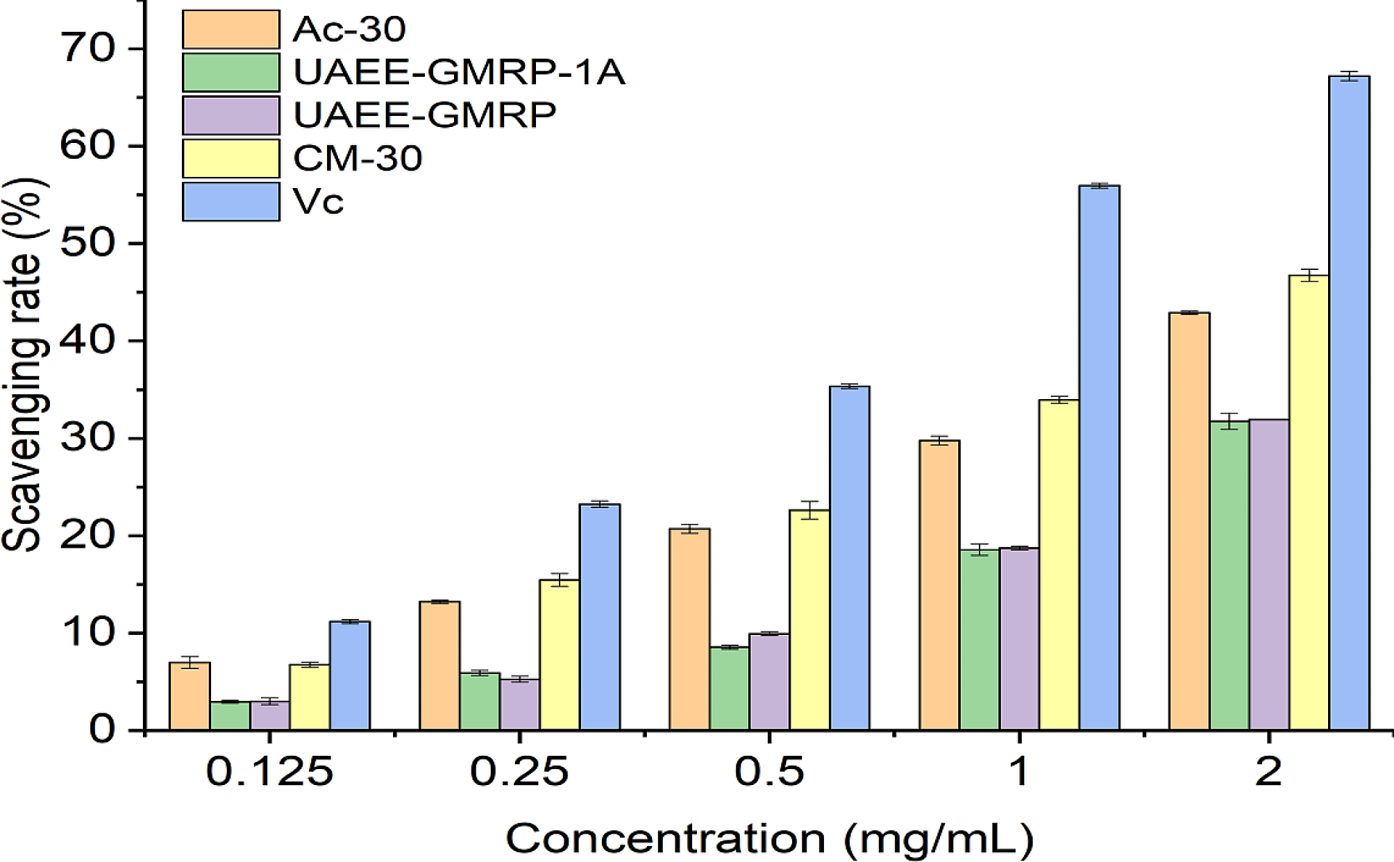
Lipid peroxidation inhibition ability

Unsaturated fatty acids and lipids in the human body often undergo oxidative degradation under high oxidation conditions, producing various harmful or even harmful free radicals. Polysaccharides can achieve the goal of clearing free radicals by inhibiting lipid peroxidation. This experiment used soy lecithin as an inducer to explore the inhibitory ability of four polysaccharides on lipid peroxidation process. The lipid peroxidation inhibition ability of the four polysaccharides is shown in Fig. 2. It can be clearly seen that their inhibition ability is V_c_> CM-30 > Ac-30 > UAEE-GMRP ≈ UAEE-GMRP-1 A. Among them, within the concentration range of 0.5 mg/mL to 2 mg/mL, the anti-lipid peroxidation ability of UAEE-GMRP and UAEE-GMRP-1 A was significantly improved compared to the lower concentration range, and there was still a good growth trend after reaching the maximum concentration. Similarly, at different polysaccharide concentrations, both polysaccharides showed similar lipid peroxidation inhibition abilities, indicating that the polysaccharide components still played the main active role in this activity experiment. However, overall, there is still a certain gap in the inhibitory effect of UAEE-GMRP and UAEE-GMRP-1 A on lipid peroxidation compared to the control group. After carboxymethylation and acetylation modification, we can see that the inhibitory ability of CM-30 and Ac-30 has been significantly improved and has exceeded the inhibitory ability of the control group by more than half when reaching the maximum concentration. The reasons for this improvement in inhibitory ability may be diverse, closely related to the number of modified substituents, substitution positions, and related reaction mechanisms. At the same time, overall, CM-30 has a stronger ability to inhibit lipid peroxidation than Ac-30, which is consistent with the previous hydroxyl radical scavenging activity experiment, indicating that the effect of carboxymethylation modification on enhancing the water solubility of polysaccharides is indeed stronger than acetylation modification. Of course, this is only one important reason, and a more comprehensive explanation requires a deeper understanding of the mechanisms of action of both. Furthermore, it can be observed that the inhibitory ability of polysaccharides on lipid peroxidation is generally inferior to their ability to scavenge hydroxyl radicals, which indirectly reflects the higher affinity of polysaccharides and their derivatives for hydroxyl radicals. The high or low affinity may be related to the unique reaction mechanisms of different in vitro antioxidant experiments. However, overall, through the above exploration experiments, it can be seen that carboxymethylation and acetylation modifications can effectively enhance the lipid peroxidation inhibition ability of polysaccharides. Among them, carboxymethylation modified products have a better enhancement effect, indicating that they have certain development potential.

## Conclusion

The hydroxyl radical scavenging ability of four polysaccharides was measured based on the Fenton reaction system, and the results showed that the scavenging rates of the four polysaccharides on hydroxyl radicals were significantly positively correlated with their concentrations. In addition, carboxymethylation and acetylation modification can effectively enhance the hydroxyl radical scavenging ability of polysaccharides, among which carboxymethylation modification has the best enhancement effect. If the polysaccharide concentration is further increased after reaching the maximum concentration, it is expected to approach or even exceed the clearance rate of the control group V_c_ in the future.

Using soybean lecithin as an inducer, the inhibitory ability of four polysaccharides on lipid peroxidation process was investigated and measured. The results showed that all four polysaccharides had a certain degree of lipid peroxidation inhibition ability. Similarly, after carboxylation and acetylation modification, the inhibitory ability of polysaccharides was effectively improved, while carboxylation modification had a better enhancement effect.

In these two antioxidant exploration experiments, it can be found that the four polysaccharides have a higher affinity for hydroxyl radicals and generally have better scavenging ability. In addition, carboxymethylation modified products showed stronger activity in both experiments, indicating that this modification method may be a polysaccharide modification method with greater potential.

## Data Availability

The datasets used and/or analysed during the current study available from the corresponding author on reasonable request.

## Abbreviations

UAEE-GMRP: Ultrasonic assisted enzyme extraction of polysaccharide from Garcinia mangostana rinds
UAEE-GMRP-1 A: Refined polysaccharide eluted from UAEE-GMRP-1 with 0.1 mol/L NaCl
CM-30: Carboxymethylated polysaccharide prepared from UAEE-GMRP was obtained under the optimal the ratio of raw material to liquid
Ac-30: Acetylated polysaccharide prepared from UAEE-GMRP was obtained under the optimal the ratio of raw material to liquid
